# A randomized controlled trial utilizing an interactive accelerometer linked to a smartphone application for enhancing physical activity and health among military employees

**DOI:** 10.3389/fspor.2025.1549980

**Published:** 2025-08-22

**Authors:** Emilia Pietiläinen, Heikki Kyröläinen, Kai Parkkola, Tiina Luukkaala, Tommi Vasankari

**Affiliations:** ^1^ Faculty of Medicine and Health Technology, Tampere University, Tampere, Finland; ^2^ Center for Military Medicine, Helsinki, Finland; ^3^ Faculty of Sport and Health Sciences, University of Jyväskylä, Jyväskylä, Finland; ^4^ Department of Leadership and Military Pedagogy, National Defence University, Helsinki, Finland; ^5^ Health Sciences, Faculty of Social Sciences, Tampere University, Tampere, Finland; ^6^ Research, Development and Innovation Center, Tampere University Hospital, Tampere, Finland; ^7^ UKK institute for Health Promotion Research, Tampere, Finland

**Keywords:** physical activity, accelerometer, mobile applications, physical fitness, cardiometabolic health, sedentary behavior

## Abstract

**Introduction:**

The primary objectives of the present individualized randomized controlled trial were to increase physical activity (PA) and improve physical fitness.

**Materials and methods:**

260 military employees around Finland participated. Two-thirds, (158), were randomized in the intervention and one-third, (101), in the control group. The intervention group used Exsed Movesense accelerometers linked to smartphones measuring PA and sleep for six months. They received feedback via a smartphone application, were encouraged to exercise during worktime for 2 hours/week, and participated in telephone counseling. The control group continued PA routines without the accelerometer or feedback. Measurements were taken at the baseline, 6-mo and 12-mo after the intervention. They included two-week RM 42-accelerometer measurements of PA, cardiometabolic biomarkers, body composition, physical fitness tests, and a questionnaire about stress and work ability for the intervention group at every point and for the control group at baseline and 12-mo. At the 6-mo, only PA was measured in the control group. Primary outcomes were changes in PA from baseline to 6-mo and 12-mo as well as changes in maximal oxygen uptake and fitness index from baseline to 12-mo. Secondary outcomes were changes in other parameters from baseline to 12-mo. The effect of the intervention on primary and secondary outcomes was analyzed using unadjusted generalised linear mixed model, accounting for a group-by-time interaction effect in all models.

**Results:**

There was no statistically significant group-by-time interaction regarding the measured parameters. However, amount the intervention group daily standing time (mean increase 18 min/day, 95% confidence interval [CI] 6–29 min/day) and maximal oxygen uptake (mean increase 2.15 ml/kg/min 95% CI 0.56–3.74 ml/kg/min) tended to increase during twelve months.

**Discussion:**

The intervention did not effectively change the primary outcomes, but showed encouraging trends and revealed the potential and challenges of the intervention developed to increase PA in a military workplace.

## 1 Introduction

Physical fitness and health are important aspects of performance and work ability (1–5). Physical activity (PA) improves physical fitness (4) and has a beneficial impact on cardiometabolic health (6–8) by improving glucose and lipid metabolism as well as reducing blood pressure (6–8). Optimal performance at work is only possible if cardiometabolic risk factors are at their lowest levels related to the workload (1). While PA has been shown to improve cardiometabolic health (6–8), it has also been shown to have a positive effect on work ability (9) and relieve stress by physiological mechanisms and by enhancing self-esteem, self-efficacy, and onés predisposition for social interactions (10). Therefore, a physically active lifestyle plays an important role in maintaining the performance of the working-age population.

The performance and work ability of military workers play an essential role in national defence, while physical fitness and health are important aspects of it (11). Soldierś daily tasks require good physical and mental performance, and professional soldiers working in the Finnish Defence Forces are obliged, according to the law, to maintain good physical fitness to meet their work requirements. While civil tasks contain mostly office or logistics work, for civilian employees, maintaining good physical fitness is optional. Still, good physical performance can be required in demanding tasks and peacekeeping operations (12).

However, PA has declined among the working-age population around the world during the last decades (13, 14). Additionally, surveys and accelerometer measurements carried out among the working-age population in Finland revealed that the volume of endurance training is not sufficient to sustain the health and fitness of the population (15, 16). Objective data recorded using accelerometers show that 76% of the day is passive time, mostly spent sitting (16). These issues challenging health and performance are seen among military personnel as well. A wide survey performed among military workers in Finland showed that only 51% of its participants exercise regularly, at least three times a week (17). Added to this, studies performed among US and German soldiers have shown a decline in performance as well as an increase in body fat over the years (18, 19).

These findings, showing the increasing inactivity of the working-age population in general (13, 14, 16) as well as of military personnel (17–19), elicit a need for interventions aimed at large populations to promote PA and thereby health and work ability (6–9). Health behavior change interventions teach their participants techniques to facilitate behavior change. Self-monitoring in combination with at least one self-regulation technique, such as goal-setting and review, have been shown to be associated with PA intervention effectiveness (20). Promising results to motivate and thus to increase PA have been gained from previous studies using tools as accelerometers combined with online feedback and smartphone techniques in adolescents and adult civilians (21–25). Furthermore, intervention utilizing smartphone-linked accelerometers serves as an opportunity for precise prevention, while the concept enables tailored, constant online feedback on the physical activity of the participants (26). Additionally, interventions taking advantage of telephone counseling combined with self-monitoring, and enabling physical activity to be performed during working hours, have been shown their effect in increasing physical activity (22, 23, 27, 28). Yet, a limited number of studies have been published utilizing smartphone-linked accelerometers in randomized controlled studies to decrease sedentary behavior (SB) or increase PA (25, 29, 30). Further, the advantages of an intervention combining smartphone-linked accelerometers, telephone counseling, and encouragement to exercise during working hours have not been investigated before among military employees.

The present study is one of the first studies that investigates the effects of long-term PA guidance on levels of PA and SB using health technology. The participants wear accelerometers and are able to monitor their PA and SB online. These types of studies using self-monitoring and goal-setting to increase PA are so far rare. Additionally, the present study aims to give new insight into this field by investigating the effects of an intervention combining an interactive accelerometer with personalized online feedback on physical activity via a smartphone application, telephone counseling, and the possibility of exercising for two hours a week during working hours among military employees. The primary objectives of the developed intervention are to increase PA and physical fitness. Additionally, the secondary objectives are to improve body composition, cardiometabolic health status, reduce stress, and improve work ability among military employees. The hypothesis is that the intervention will increase daily PA; decrease SB; and improve physical fitness.

## 2 Materials and methods

### 2.1 Intervention protocol

Permission to perform our study was granted by the local ethics committee of the Tampere University Hospital (R16189) and Defence Command Finland (AN8355, 1367/12.04.01/2015; AN8355, 2055/09.05/2022). The six-month intervention and its subsequent six-month follow-up protocol, as well as the timeline of the measurements performed in different phases of the study, are illustrated in Table 1. The intervention group started their intervention six months after the baseline measurements. The intervention concentrating on PA lasted six months, during which time the intervention group participants wore an interactive accelerometer (ExSed Movesense, Suunto, Finland) that was linked to a smartphone application (ExSed, UKK, Terveyspalvelut Oy, Tampere, Finland) and, further, to a cloud service. The accelerometers were checked to function similarly before giving them to the intervention group participants. The participants received information on their accumulated daily PA, SB, standing, and quality of sleep via the smartphone application. The accelerometer was downloaded with ExSed algorithms with a mean amplitude deviation and angle for posture estimation that were developed and validated at the UKK Institute (Tampere, Finland) (31–33). The accelerometer was worn on the hip during waking hours and on the wrist during sleep. It transmitted the PA and sleep data to the ExSed application via Bluetooth. The application provided constant feedback on PAs (light PA [LPA], moderate-to-vigorous PA [MVPA]), steps, standing, as well as on the quality of sleep (total sleep time, restless sleep time, restful sleep time). The recorded data was transmitted and saved to a cloud service (34). The application set daily goals for PA for each day, which were in line with the national recommendations (35, 34). The personal guidance and feedback were transmitted as illustrative histograms (graphical columns that illustrated participantś activity and achievement of their daily goals). Participants could observe online the accumulation and sufficiency of their daily PA by monitoring the ExSed application whenever most convenient (34). After this six-month intervention period, the intervention group participants returned the accelerometers to the UKK institute.

**Table 1:** The intervention protocol and the measurements performed in different phases of the study.

Measurements	Baseline	Intervention (0–6 months)	6-month follow-up		12-month follow-up	
	All participants (*n* = 260)	Intervention group (*n* = 158)	Intervention group (*n* = 94)	Control group (*n* = 63)	Intervention group (*n* = 79)	Control group (*n* = 59)
Questionnaire	X		X		X	X
Body composition	X		X		X	X
RM42 accelerometer measurements	X		X	X	X	X
Fitness tests	X		X		X	X
Cardiometabolic biomarkers	X		X		X	X
Movesense accelerometer measurements and smartphone app feedback		X				
Telephone counseling session		X				

The intervention group participants also received telephone counseling about their PA and exercise habits once during the six-month intervention period. The counseling session could be scheduled earliest a month from the beginning of the intervention and, at the latest, a month before the end of the intervention. During the counseling session (lasting 10 minutes), the participants were interviewed about their PA habits and the factors hindering these habits. The physical education instructor gave individualized feedback to the participants and encouraged the participants to increase their PA and decrease their SB if the PA was not sufficient. If the participants’ PA was at a good level, they were encouraged to continue their exercise and PA routines as they had by far. The telephone counseling sessions were carried out by two physical education instructors and a research physician using a pre-planned interview sheet. Moreover, the participants of the intervention group were also encouraged to use the existing benefit to exercise for two hours a week during working hours from the beginning of the intervention.

The control group continued their normal exercise routines without an accelerometer or feedback, while the intervention was ongoing among the intervention group participants. This possibility to exercise during working hours is offered to all the employees working in the FDF under the existing FDF guidelines, therefore, the control group participants could use the possibility to exercise during working hours, but they were not separately encouraged to do so.

### 2.2 Sample size calculations

Before the intervention, the sample size was calculated using the participantś fitness indices. The calculations assumed that the fitness indices would increase by 0.14 for male soldiers; 0.45 for female soldiers; 0.34 for male civilian employees; and 0.24 for female civilian employees. For the evaluation of physical fitness, the appropriate number of study participants was approximately 175 soldiers and 99 civilian employees [G*Power 3.1.9.2, Wilcoxon signed-rank test (matched pairs), correlation between groups assumption *r* = 0.80] (36).

**Figure 1:**
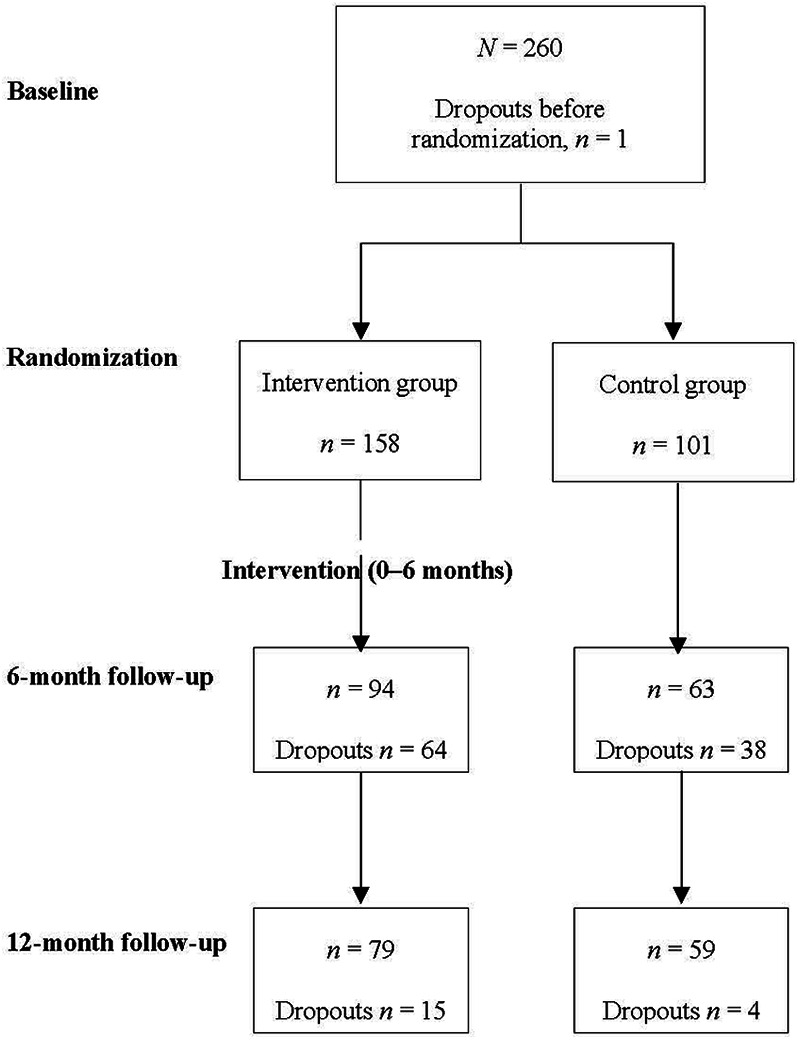
Dropout chart.

Drop-outs in different phases are presented in Figure 1. The reasons for dropping out included changing workplace to a new location, attending a crisis management mission, the loss of the accelerometer, the dysfunction of the accelerometer, personal reasons, the loss of motivation, health reasons, the lack of time, the long study duration, feeling uncomfortable using the accelerometer and not gaining any interesting information.

In the *post hoc* power analysis, the effect size was calculated using the means and standard deviations of PA and physical fitness test results at the twelve-month follow-up. Then, the mean and standard deviation of the fitness index would be 3.7 (SD 1.53) in the intervention group and 3.3 (SD 1.17) in the control group (Supplementary Table A), with an allocation ratio 2:1. The effect size would be *d* = 0.294 (small), and the sample size needed would be 275 participants in the intervention group and 137 participants in the control group (calculated using G*Power 3.1.9.7).

### 2.3 Randomization

After the baseline measurements, the participants were randomized into an intervention group and a control group using a randomization questionnaire. The intervention was implemented according to the intention-to-treat principle. Two-thirds of the participants were assigned to the intervention group and one-third to the control group. The randomization questionnaire asked for personal information to create homogenous groups according to age, gender, working status (civilian employee vs. soldier) and PA as during how many days a week they performed moderate-intensity physical activity (options were: 1 [“Not at all”], 2 [“Only light intensity physical activity at least on one day a week”], 3 [“Once a week”], 4 [“Twice a week”], 5 [“Three times a week”], 6 [“At least four times a week”]). The type of smartphone or other smart device that the participants used was asked about to confirm that the participants in the intervention group had a device that supported the application used in the intervention. The participants had an ID linked to their questionnaire information, which ensured that the results of the participant could not be identified with a certain individual. The data were expressed in numeric form: age (2 [ages 20–29], 3 [ages 30–39], 4 [ages 40–49], 5 [ages 50 and over]), gender (1 [man], 2 [woman]), and PA (1–6 according to the answer choice). The research physician gave the randomization questionnaires to the participants, collected the questionnaires, created their IDs, and exported the questionnaire information into the excel sheet. The results from the questionnaires, exported into the excel sheet, including participant IDs, were sent to the UKK institute and manually randomized by a separate researcher, ensuring during the process that the profile of both groups would appear similar. Finally, 158 of the participants were randomized to the intervention group and 101 to the control group.

### 2.4 Measurements

All participants were measured at three different time points during the study: at the baseline, after the intervention at the six-month follow-up, and at the twelve-month follow-up (six months from the end of the intervention). The performed measurements were similar for all participants at the baseline and twelve-month follow-up points, but only intervention group participants performed all the measurements at the six-month follow-up point. The control group participated only PA and SB measurements at six-month follow-up point. At the beginning of the study, all the participants participated in baseline measurements, which were carried out over a two-month period. The baseline measurements included measurements of PA with an RM42 accelerometer (UKK Terveyspalvelut Oy, Tampere, Finland) for two weeks, body composition measurements, blood samples for cardiometabolic biomarkers, fitness tests, and a questionnaire inquiring about experienced stress and working ability. The intervention group participants were measured again at the six-month and twelve-month follow-up points, as at the baseline. At the six-month follow-up point, the intervention had ended, and the intervention group had returned their Movesense accelerometers to the UKK institute. Between the six-month and twelve-month follow-up points, the intervention group continued their normal routines without the accelerometer or feedback.

At the six-month follow-up point, the control group only performed PA measurements using an RM42 accelerometer for two weeks to examine whether there were any changes in their PA as a result of participating in the study without participating in the intervention. The last measurements, to study the sustainability of the intervention, were performed at the twelve-month follow-up point and these measurements were the same as at the baseline for the control group participants, as they were for the intervention group participants.

The accelerometer (UKK RM42; UKK Terveyspalvelut Oy) measurements for two weeks were used to analyze the PA of the participants at the baseline, at the six-month follow-up, and at the twelve-month follow-up. The accelerometers were worn on a belt on the hip during waking hours. As the accelerometer is not water-resistant, it had to be taken off when showering and during water activities. After the two-week measurement period, the accelerometers were sent to the UKK Institute (Tampere, Finland), where the recorded data was analyzed. All participants received results and feedback from these accelerometer measurements from all time points only after the twelve-month follow-up measurements.

The device measured the acceleration in three orthogonal x, y, and z directions at a sampling rate of 100 Hz, and the resultant was established using these three components. Added to this, the mean amplitude deviation of the resultant was analyzed in 6 s epoch lengths (33). PA was categorized into *light, moderate,* and *vigorous* activity according to metabolic equivalents (METs). A resting metabolic rate of 1 MET corresponds to a 3.5 ml/kg/min oxygen consumption rate (32, 33). Time spent in a sitting and reclining position was considered as SB. SB has an energy expenditure of ≤1.5 MET, LPA has an energy expenditure of <3 MET, and MVPA has an energy expenditure of ≥3 MET. The oxygen cost of movement (ml/kg/min) was calculated for each stage as the ratio of measured oxygen consumption (ml/kg/min) to known speed (km/min) as described by Vähä-Ypyä et al. (32). Steps were identified using the magnitude of the horizontal component of the dynamic acceleration (37). Standing still could be separated from sitting or lying with 100% accuracy, and it was analyzed separately (32, 33, 31).

The daily step count, length of total LPA, MVPA, standing time, and SB were analyzed as well as accumulated PA bouts containing both LPA and MVPA, and stationary bouts. Stationary time was referred to as time spent lying, reclining, or sitting, while standing was analyzed separately (31, 38). The daily averages for total PA accumulated from bouts lasting 0–3 min, 3–10 min and longer than 10 min–as well as total stationary time accumulated from bouts lasting 0–20 min, 20–60 min and longer than 60 min–were also calculated for each participant. Participants having data of 10 hours from at least four days were included for further analyses. The group values were analyzed using the mean values for each participant.

The body composition of the participants was measured by using body mass, body height, body mass index (BMI), fat percentage (FAT%), and waist circumference. The measurements were performed in the morning after at least two hours of fasting. The segmental multifrequency bioimpedance analysis assessment (InBody 720, Biospace Co Ltd., Seoul, Korea) was used to measure body mass, BMI, and FAT%. A tape measure was used to measure the waist circumference in the midline between the lowest rib and iliac crest after exhaling.

Selected cardiometabolic biomarkers were used to evaluate the cardiometabolic health of the participants. The blood samples for the cardiometabolic biomarkers were collected after twelve hours of fasting. Serum fasting total cholesterol, low-density lipoprotein cholesterol (LDL), high-density lipoprotein cholesterol (HDL), and triglycerides were analyzed to assess lipid metabolism. Fasting blood glucose (gluc), glycated hemoglobin (HbA1c), and insulin (INS) were analyzed to evaluate glucose metabolism.

HDL, triglycerides, gluc, and HbA1c were analyzed using a Konelab 20 XTi device (Thermo Electron Co., Vantaa, Finland), and an isolated LDL fraction was used for the direct measurement of LDL (the Enzymatic Colorimetric Determination of Serum Cholesterol method). The sensitivity for gluc and HbA1c are 0.1 mmol/L and 0.03 mmol/L, and the intra-assay coefficients of variance are 1.0% and 8.6% respectively. The ranges for the total cholesterol, triglyceride, HDL, and LDL assays vary from 0.1 to 15, from 0.09 to 11, from 0.04 to 2.84, and from 0.3 to 8.9 mmol/L, respectively. The intra-assay coefficients of variance are 1.1% for total cholesterol, 1.0% for triglycerides, 3.4% for LDL and 0.5% and for HDL. INS was analyzed using chemical luminescence techniques (Immulite 2000, Siemens Healthcare Diagnostics, Camberley, UK) with an assay sensitivity of 2 mIU/L and an inter-assay coefficient of variation of 5.1%.

Endurance and muscle fitness tests were used to evaluate the physical fitness of the participants. The endurance test assessed the aerobic capacity of the participants, and the participants performed a twelve-minute running test (39), or a cycle ergometer test (40), or a UKK 2-km walking test (41) as an endurance test. Every participant had to use the same test at every measurement point. Maximal oxygen uptake (VO_2_max) was used to express the maximal aerobic capacity. The muscle fitness test consisted of a maximal standing long jump (SLJ), evaluating the maximal power production of the lower extremities, as well as one minute of sit-ups, and one minute of push-ups, assessing the dynamic muscle endurance capacity of the trunk and upper extremities (42). According to the results of an endurance test and a muscle strength test, participantś endurance and muscle strength indices were determined using age-dependent reference values. The fitness index was calculated as an average of the endurance and muscle strength indices (12).

The work ability of the participants was analyzed with the statement and question “Work ability at the moment compared with work ability at its highest during onés career. It is assumed that your work ability gains 10 points at its highest. What score would you give your current work ability on a scale of 0–10 (a zero-score meaning “total inability to work”)?” The options ranged from 0 meaning the total inability to work, to 10, meaning work ability at its highest (43). This single question on work ability is widely used and validated in evaluating work ability. A high correlation has been detected between this single question on work ability and work ability index (44–46). Further, this single question on work ability has been shown to be as valid as the work ability index (47) in evaluating work ability of employees.

Experienced stress was evaluated with a statement and question from Occupational Stress Questionnaire “Stress is an emotional state where a person feels tense, restless, anxious, or nervous, and/or the person has difficulty sleeping because of troubling thoughts. Are you experiencing this kind of stress at the moment?” The answering options were 1 (“Not at all”), 2 (“I'm only experiencing this a little”), 3 (“I'm experiencing this somewhat”, 4 (“I'm experiencing this rather a lot”) and 5 (“I'm experiencing this a lot”)­ (48). The Occupational Stress Questionnaire is a widely used method in occupational health to assess characteristics and stress factors of work and stress reactions of employees in Finnish companies (49, 50). The questionnaire has been investigated in several research projects of the Finnish Institute of Occupational Health since 1977 (49). The validity of the stress question has been studied, and there is an association between psychological symptoms and mental resources (51).

### 2.5 Primary and secondary outcomes

The primary outcomes of the study were differences between the groups in changes in PA measured with the RM42 accelerometer from baseline to six-month and twelve-month follow-ups, as well as differences between the groups in changes in physical fitness from baseline to twelve-month follow-up. More specifically, these included MVPA, LPA, SB, standing, and daily step count from baseline to six- and twelve-month follow-ups, as well as changes in VO_2_max and fitness index from baseline to twelve-month follow-up.

Secondary outcomes of the study were differences between the groups in changes in body composition, cardiometabolic biomarkers, muscular fitness test results, experienced stress, and work ability from baseline to the twelve-month follow-up. These included changes in BMI, FAT%, waist circumference, total cholesterol, HDL, LDL, triglycerides, HbA1c, gluc, INS, push-ups, sit-ups, and SLJ from baseline to twelve-month follow-up. Further, secondary outcomes included differences between the groups in changes in PA accumulated from bouts lasting 0–3 min, 3–10 min, and longer than 10 min, as well as stationary time accumulated from bouts lasting 0–20 min, 20–60 min and longer than 60 min from baseline to the six-month- and twelve-month follow-ups (36).

### 2.6 Statistical analyses

Parameters of PA, body composition, cardiometabolic biomarkers, fitness test results, experienced stress, and work ability are described by mean with 95% confidence interval for mean. Two-sided *p*-values under 0.05 were considered as statistically significant. Statistical analyses were carried out with SPSS statistical software (SPSS, IBM, Armonk, New York, USA) versions 29.0.1.0. Unadjusted effects of the changes in the measured parameters were modelled as normally distributed, distributed over time (baseline, six-, and twelve- months) using Generalized Linear Mixed Models. Group-by-time interaction effect was included in all models. Participants created potential sources of variation, and therefore, this subject-specific effect was included as a random effect in the model (52). Explanatory factors were modelled as fixed effects. The Generalized Linear Mixed Model was applied with the R statistical software package (version 4.4.2, Ime4 function, R Core Team [2024]. R: A language and environment for statistical computing. R Foundation for Statistical Computing, Vienna, Austria. URL https://www.R-project.org/).

## 3 Results

### 3.1 Participants

Participants were recruited from six military brigades around Finland by the research physician with help from brigadeś physical education officers. They were soldiers and civilian employees working in the Finnish Defence Forces (FDF). The participants had to be aged between 19 and 60 years old and eligible to perform the annual fitness tests of the FDF. An occupational physician determined if the health of the participant allowed her or him to perform the fitness tests (36). The participants were given information about the study, and they gave their written informed consent to participate.

Altogether, 260 military employees participated in the study. Characteristics of the participants are presented in Table 2. Altogether 108 (68%) of the 158 participants in the intervention group were soldiers, and 113 (72%) were men. In the control group, 77 (76%) of the participants were soldiers, and 81 (80%) were men.

**Table 2:** Characteristics of the intervention and control group participants.

Characteristics of the participants	Intervention (*n* = 158)	Controls (*n* = 101)
	*n* (%)	*n* (%)
Men	113 (71.5)	81 (80.2)
Soldiers	108 (68.4)	77 (76.2)
	Mean ± SD	Mean ± SD
Age (years)	42 (8)	41 (9)
Sedentary behavior (min/day)	560 (78)	556 (93)
Standing (min/day)	99 (41)	112 (50)
Light physical activity (min/day)	221 (59)	220 (57)
Moderate-to-vigorous physical activity (min/day)	49 (20)	52 (23)
Steps (number/day)	6,955 (2,027)	7,237 (2,250)
Maximal oxygen uptake (ml/kg/min)	42.7 (9.5)	43.2 (10.7)
Fitness index	3.54 (1.16)	3.53 (1.05)
Body mass index (kg/m^2)^	27.2 (4.4)	27.0 (4.2)
Fat percentage (%)	24.7 (9.7)	23.3 (9.2)
Waist circumference (cm)	93.0 (12.4)	93.0 (11.6)
Total cholesterol (mmol/L)	4.95 (0.89)	4.88 (0.75)
High-density lipoprotein cholesterol (mmol/L)	1.47 (0.40)	1.53 (0.45)
Low-density lipoprotein cholesterol (mmol/L)	3.28 (0.84)	3.16 (0.76)
Triglycerides (mmol/L)	1.17 (0.83)	1.13 (0.58)
Glycated hemoglobin (mmol/mol)	34.7 (5.4)	34.3 (3.8)
Fasting glucose (mmol/L)	5.4 (0.9)	5.3 (0.5)
Insulin (mIU/ml)	6.0 (7.2)	5.3 (5.1)
Push-ups (reps/min)	37 (14)	36 (13)
Sit-ups (reps/min)	37 (13)	39 (11)
Standing long jump (m)	2.08 (0.43)	2.06 (0.38)
Work ability	8.3 (1.3)	8.2 (1.6)
Stress	2.3 (1.0)	2.6 (1.0)
Stationary time in 0–20 min bouts (min/day)	356 (58)	358 (56)
Stationary time in 20–60 min bouts (min/day)	210 (52)	217 (63)
Stationary time in longer than 60 min bouts (min/day)	94 (52)	93 (50)
Physical activity in 0–3 min bouts (min/day)	150 (32)	151 (31)
Physical activity in 3–10 min bouts (min/day)	53 (18)	53 (19)
Physical activity in longer than 10 min bouts (min/day)	66 (35)	67 (35)

### 3.2 Physical activity (PA)

Changes in the PA measurements during the one-year follow-up are presented in Table 3. There was no statistically significant group-by-time interaction in the PA parameters. Only the standing time tended to increase (mean increase 18, 95% CI 6–29 min/day) among the intervention group participants during the twelve-month follow-up compared to the control group participants (mean decrease −4 95% CI −23–15 min/day), among whom the standing time decreased.

**Table 3:** Changes (Δ) in physical activity in measurements from baseline (BL) to six-month and twelve-month (mo) follow-ups.

Physical activity parameters	Intervention group			Control group			t-values (and *p*-values) for differences				
	BL (*n* = 146)	ΔBL to 6-mo (*n* = 68)	ΔBL to 12 mo (*n* = 53)	BL (*n* = 93)	ΔBL to 6-mo (*n* = 47)	ΔBL to 12-mo (*n* = 43)	Group effect	Time effect (6-mo)	Time effect (12-mo)	Group × time interaction (6-mo)	Group × time interaction (12-mo)
	Mean (95% CI)	Mean (95% CI)	Mean (95% CI)	Mean (95% CI)	Mean (95% CI)	Mean (95% CI)	t-value (*p*-value)	t-value (*p*-value)	t-value (*p*-value)	t-value (*p*-value)	t-value (*p*-value)
SB (min/day)	560 (547–573)	−30 (−50 to −10)	−5 (−30–20)	556 (536–575)	−22 (−48–4)	11 (−18–40)	0.249 (0.803)	−2.544 (0.012)	0.432 (0.666)	−0.045 (0.964)	−1.139 (0.256)
Standing (min/day)	99 (92–106)	5 (−5–14)	18 (6–29)	112 (101–122)	3 (−11–17)	−4 (−23–15)	−1.853 (0.065)	1.1316 (0.189)	0.039 (0.969)	−0.058 (0.954)	1.924 (0.056)
LPA (min/day)	221 (211–232)	−1 (−15–12)	−14 (−26 to −1)	220 (208–231)	10 (−5–24)	−15 (−31–1)	0.473 (0.636)	1.499 (0.135)	−1.476 (0.141)	−1.011 (0.313)	−0.208 (0.835)
MVPA (min/day)	49 (45–52)	6 (1–11)	−4 (−9–2)	52 (46–56)	8 (−1–16)	−3 (−9–4)	−0.996 (0.320)	2.553 (0.011)	−0.796 (0.427)	−0.202 (0.840)	−0.038 (0.970)
Steps (number/day)	6,955 (6,623–7,287)	307 (−202–817)	−265 (−752–223)	7,237 (6,774–7,701)	730 (70–1,391)	−322 (−957–312)	−0.969 (0.333)	2.545 (0.012)	−0.959 (0.338)	−0.673 (0.502)	0.275 (0.784)
St 0–20 (min/day)	356 (346–366)	−18 (−35 to −2)	−8 (−23–6)	358 (347–369)	−6 (−21–9)	−19(−34 to −3)	−0.001 (0.999)	−0.220 (0.826)	−1.252 (0.212)	−0.987 (0.324)	0.019 (0.985)
St 20–60 (min/day)	210 (101–219)	−3 (−15–10)	9 (−5–23)	217 (204–231)	−11 (−27–5)	6 (−12–24)	−1.151 (0.251)	−2.2025 (0.044)	0.033 (0.974)	1.257 (0.210)	0.689 (0.491)
St 60+ (min/day)	94 (85–103)	−5 (−18–9)	12 (−1–25)	93 (82–103)	−2 (−18–14)	20 (1–38)	−0.108 (0.914)	−0.762 (0.447)	2.064 (0.040)	−0.448 (0.655)	−0.990 (0.323)
PA 0–3 (min/day)	150 (145–156)	−7 (−14–1)	−5 (−12–2)	151 (144–158)	−1 (−10–8)	−8 (−17 to −0.5)	0.111 (0.912)	0.112 (0.911)	−1.336 (0.183)	−1.044 (0.297)	−0.023 (0.981)
PA 3–10 (min/day)	53 (50–57)	2 (−3–6)	−4 (−8–1)	53 (48–57)	1 (−4–7)	−5 (−11–1)	0.369 (0.712)	0.431 (0.667)	−2.114 (0.036)	0.298 (0.766)	0.163 (0.871)
PA 10+ (min/day)	66 (60–72)	10 (1–19)	´−9 (−18–1)	67 (59–75)	17 (3–31)	−4 (−15–6)	−0.198 (0.843)	2.959 (0.003)	−0.997 (0.320)	−0.434 (0.665)	−0.055 (0.956)

CI, confidence interval; SB, sedentary behaviour; LPA, light physical activity; MVPA, moderate to vigorous physical activity; St 0–20, Stationary time in 0–20 min bouts; St 20–60, stationary time in 20–60 min bouts; St 60+, stationary time longer than 60 min bouts; PA 0–3, physical activity in 0–3 min bouts; PA 3–10, physical activity in 3–10 min bouts; PA 10+, physical activity in longer than 10 min bouts. Group (Intervention vs. control) and time (6-months vs. BL and 12 months vs. BL) effects and group-by-time interactions were modelled using generalized linear mixed models.

SB decreased in the intervention group during the follow-up, but not in the control group. The overall effect of stationary time accumulated from bouts lasting 0–20 minutes and PA accumulated from bouts lasting 0–3 min/day was decreasing in both groups. The direction of other PA parameters varied during follow-up.

### 3.3 Body composition

Changes in body composition during the one-year follow-up are presented in Table 4. There was no group-by-time interaction in body composition between the intervention and control groups during the twelve-month follow-up. When observing changes in body composition over time, FAT% decreased from the baseline to the end of follow-up in both groups. Both BMI and waist circumference decreased in the intervention group but increased in the control group, but still without a statistically significant interaction effect. The magnitude of these changes is described in detail in Table 4.

**Table 4:** Changes (Δ) in body composition from baseline (BL) to six-month and twelve-month (mo) follow-ups.

Body composition parameters	Intervention group			Control group		t-values (*p*-values) for differences			
	BL	ΔBL to 6-mo	ΔBL to 12-mo	BL	ΔBL to 12-mo	Group effect	Time effect (6- mo)	Time effect (12- mo)	Group × time interaction (12-mo)
	Mean (95% CI)	Mean (95% CI)	Mean (95% CI)	Mean (95% CI)	Mean (95% CI)	t-value (*p*-value)	t-value (*p*-value)	t-value (*p*-value)	t-value (*p*-value)
Body mass index (kg/m²)	*n* = 154	*n* = 72	*n* = 50	*n* = 100	*n* = 34	0.358 (0.721)	0.538 (0.591)	0.729 (0.467)	−1.121 (0.264)
	27.2 (26.5–27.9)	0.1 (−0.2–0.4)	−0.2 (−0.6–0.3)	27.0 (26.1–27.8)	0.2 (−0.2–0.5)				
Fat percentage (%)	*n* = 144	*n* = 68	*n* = 47	*n* = 92	*n* = 31	1.247 (0.213)	−2.924 (0.004)	−0.319 (0.750)	−1.373 (0.172)
	24.7 (23.1–26.2)	−1.1 (−1.8 to −0.3)	−1.1 (−2.0 to −0.1)	23.3 (21.3–25.2)	−0.1 (−1.1–1−0)				
Waist circumference (cm)	*n* = 143	*n* = 27	*n* = 36	*n* = 93	*n* = 21	−0.115 (0.908)	−0.217 (0.829)	0.225 (0.823)	−0.539 (0.591)
	93.0 (90.9–95.1)	−0.1 (−2.6–2.4)	−0.4 (−2.8–2.1)	93.0 (90.5–95.4)	0.4 (−1.1–1.9)				

CI, confidence interval. Group (Intervention vs. control) and time (6-mo. vs. BL and 12 mo. vs. BL) effects and group-by-time interactions were modelled using generalized linear mixed models. No results from the control group, while their body composition was not measured at the six-month follow-up point.

### 3.4 Cardiometabolic biomarkers

Changes in cardiometabolic biomarkers during the one-year follow-up are presented in Table 5. There was no group-by-time interaction in the cardiometabolic biomarkers between the intervention and control groups during the twelve-month follow-up. Still, total cholesterol and LDL decreased in the intervention group but increased in the control group. HDL tended to decrease and triglycerides increase in both groups. All results measuring glucose metabolism tended to increase in both intervention and control groups. The magnitude of these changed is described in detail in Table 5.

**Table 5:** Changes (Δ) in cardiometabolic biomarkers from baseline (BL) to six-month and twelve-month (mo) follow-ups.

Cardiometabolic biomarkers	Intervention group			Control group		t-values (*p*-values) for differences			
	BL	ΔBL to 6-mo	ΔBL to 12-mo	BL	ΔBL to 12-mo	Group effect	Time effect (6- mo)	Time effect (12-mo)	Group × time interaction (12-mo)
	Mean (95% CI)	Mean (95% CI)	Mean (95% CI)	Mean (95% CI)	Mean (95% CI)	t-value (*p*-value)	t-value (*p*-value)	t-value (*p*-value)	t-value (*p*-value)
Total cholesterol (mmol/L)	*n* = 139	*n* = 73	*n* = 54	*n* = 91	*n* = 43	0.393 (0.695)	−1.447 (0.149)	1.191 (0.235)	−1.601 (0.111)
	4.95 (4.80–5.11)	−0.14 (−0.33–0.04)	−0.14 (−0.34–0.07)	4.88 (4.72–4.04)	0.06 (−0.17–0.29)				
High-density lipoprotein cholesterol (mmol/L)	*n* = 139	*n* = 73	*n* = 54	*n* = 91	*n* = 43	−1.084 (0.280)	−2.081 (0.039)	−2.189 (0.030)	0.164 (0.870)
	1.47 (1.40–1.54)	−0.07 (−0.13–0.01)	−0.06 (−0.14–0.01)	1.53 (1.43–1.63)	−0.08 (−0.13 to −0.02)				
Low-density lipoprotein cholesterol (mmol/L)	*n* = 139	*n* = 73	*n* = 54	*n* = 91	*n* = 43	0.992 (0.322)	−1.011 (0.313)	1.057 (0.292)	−1.392 (0.165)
	3.28 (3.13–3.42)	−0.08 (−0.23–0.07)	−0.05 (−0.24–0.14)	3.16 (3.00–3.32)	0.06 (−0.16–0.28)				
Triglycerides (mmol/L)	*n* = 139	*n* = 73	*n* = 54	*n* = 91	*n* = 43	−0.151 (0.880)	−0.324 (0.746)	1.393 (0.165)	−0.865 (0.388)
	1.17 (1.03–1.32)	0.01 (−0.09–0.10)	<0.01 (−0.13–0.13)	1.13 (1.00–1.25)	0.19 (0–0.37)				
Glycated hemoglobin (mmol/mol)	*n* = 138	*n* = 73	*n* = 57	*n* = 89	*n* = 44	0.319 (0.750)	14.788 (<0.001)	11.446 (<0.001)	0.165 (0.869)
	34.7 (33.7–35.7)	5.8 (4.8–6.7)	6.0 (5.0–7.0)	34.3 (33.5–35.2)	5.7 (4.9–6.5)				
Fasting glucose (mmol/L)	*n* = 122	*n* = 63	*n* = 51	*n* = 84	*n* = 40	1.008 (0.314)	2.330 (0.021)	−0.015 (0.988)	0.962 (0.337)
	5.4 (5.2–5.6)	0.22 (−0.05–0.49)	0.11 (−0.04–0.26)	5.3 (5.1–5.4)	0.02 (−0.14–0.19)				
Insulin (mIU/ml)	*n* = 139	*n* = 73	*n* = 54	*n* = 91	*n* = 43	0.076 (0.940)	1.310 (0.192)	−0.124 (0.902)	1.142 (0.255)
	6.0 (4.8–7.3)	1.10 (−0.66–2.88)	0.97 (−0.66–2.59)	5.3 (4.2–6.4)	1.37 (−0.21–2.94)				

CI, confidence interval. Group (intervention vs. control) and time (6-mo vs. BL and 12 mo vs. BL) effects and group-by-time interactions were modelled using generalized linear mixed models. No results from the control group, while laboratory tests were not taken from them at the six-month follow-up point.

### 3.5 Fitness tests

Changes in the fitness test results during the one-year follow-up are presented in Table 6. There was no statistically significant group-by-time interaction in the fitness test results between the intervention and control groups during the twelve-month follow-up. However, the VO_2_max tended to increase among the intervention group (mean increase 2.15 CI 95% 0.56–3.74 ml/kg/min) participants compared to those in the control group (mean decrease −0.71 CI 95% −3.880–2.37), but the group-by-time interaction did not reach full statistical significance (*t* = 2.009, *p* = 0.052). The trends in fitness index and push-ups results were increasing in both intervention and control groups, but the results of other studied parameters varied during the follow-up. The magnitude of these changes is described in detail in Table 6.

**Table 6:** Changes (Δ) in fitness test results from baseline (BL) to six-month and twelve-month (mo) follow-ups.

Fitness test	Intervention group			Control group		t-values (*p*-values) for differences			
	BL	ΔBL to 6-mo	ΔBLto12-mo	BL	ΔBL to 12-mo	Group effect	Time effect (6- mo)	Time effect (12-mo)	Group × time interaction (12- mo)
	Mean (95% CI)	Mean (95% CI)	Mean (95% CI)	Mean (95% CI)	Mean (95% CI)	t-value (*p*-value)	t-value (*p*-value)	t-value (*p*-value)	t-value (*p*-value)
Fitness index	*n* = 127	*n* = 9	*n* = 13	*n* = 87	*n* = 8	0.003 (0.974)	0.923 (0.363)	1.065 (0.295)	0.638 (0.528)
	3.54 (3.33–3.75)	0.10 (−0.20–0.40)	0.33 (0.12–0.53)	3.53 (3.30–3.76)	0.24 (−0.06–0.56)				
Maximal oxygen uptake (ml/kg/min)	*n* = 138	*n* = 10	*n* = 17	*n* = 95	*n* = 10	−0.524 (0.601)	1.246 (0.220)	−0.572 (0.571)	2.009 (0.052)
	42.7 (41.0–44.3)	1.79 (−0.87–4.45)	2.15 (0.56–3.74)	43.2 (11.0–45.4)	−0.71 (−3.880–2.37)				
Push-ups (rep/min)	*n* = 139	*n* = 23	*n* = 14	*n* = 90	*n* = 8	0.182 (0.856)	0.400 (0.691)	1.154 (0.254)	0.070 (0.944)
	37 (34–39)	0.48 (−3.11–4.07)	2.86 (0.27–5.45)	36 (33–40)	3.50 (−0.92–7.92)				
Sit-ups (rep/min)	*n* = 140	*n* = 24	*n* = 14	*n* = 90	*n* = 8	−1.182 (0.238)	−0.764 (0.449)	−1.122 (0.268)	0.947 (0.348)
	37 (34–39)	−0.5 (−2.3–1.2)	0.5 (−2.4–3.4)	38 (36–42)	−1.4 (−3.8–1.0)				
Standing long jump (m)	*n* = 139	*n* = 24	*n* = 15	*n* = 90	*n* = 8	0.350 (0.726)	−0.085 (0.932)	0.042 (0.966)	−0.160 (0.873)
	2.08 (2.00–2.16)	0.01 (−0.10–0.12)	−0.03 (−0.08–0.03)	2.06 (1.98–2.15)	0.001 (−0.06–0.06)				

CI, confidence interval. Group (Intervention vs. control) and time (6-mo vs. BL and 12 mo vs. BL) effects and group-by-time interactions were modelled using generalized linear mixed models. No results from the control group, while they did not perform the fitness tests at the six-month follow-up point.

### 3.6 Stress and work ability

Changes in stress and work ability during the one-year follow-up are presented in Table 7. There was less stress among the intervention group than the control group. During the one-year follow-up, stress was perceived to be higher, and work ability lower in both the intervention and control groups. The magnitude of these changes is described in detail in Table 7.

**Table 7:** Changes (Δ) in experience stress and work ability from baseline (BL) to six-month and twelve-month (mo) follow-ups.

Stress and work ability	Intervention group			Control group		t-values (*p*-values) for differences			
	BL	ΔBL to 6-mo	ΔBL to 12-mo	BL	ΔBL to 12-mo	Group effect	Time effect (6- mo)	Time effect (12-mo)	Group × time interaction (12- mo)
	Mean (95% CI)	Mean (95% CI)	Mean (95% CI)	Mean (95% CI)	Mean (95% CI)	t-value (*p*-value)	t-value (*p*-value)	t-value (*p*-value)	t-value (*p*-value)
Stress	*n* = 131	*n* = 45	*n* = 60	*n* = 75	*n* = 40	−2.221 (0.027)	2.418 (0.017)	−0.037 (0.971)	1.017 (0.310)
	2.28 (2.12–2.45)	0.29 (0.01–0.57)	0.18 (−0.03–0.40)	2.57 (2.34–2.80)	0.03 (−0.28–0.33)				
Work ability	*n* = 130	*n* = 45	*n* = 60	*n* = 75	*n* = 40	1.244 (0.215)	−0.896 (0.372)	−1.877 (0.062)	1.020 (0.309)
	8.37 (8.14–8.60)	−0.09 (−0.41–0.23)	−0.02 (−0.26–0.23)	8.16 (7.80–8.52)	−0.30 (−0.64–0.04)				

CI, confidence interval. Group (intervention vs. control) and time (6-mo vs. BL and 12 mo vs. BL) effects and group-by-time interactions were modelled using generalized linear mixed models. No results from the control group, while they did not answer the questionnaire at the six-month follow-up point.

## 4 Discussion

The implemented intervention tended to increase daily standing time among the intervention group in the long term, even though there was no intervention group interaction regarding the PA or SB from the baseline to the six-month or twelve-month follow-ups. Previously, interventions targeted to reduce SB have shown reductions in sitting time largely achieved by standing (53). However, during the time interval from the baseline to the twelve-month follow-up, there was a reduction in MVPA and LPA in addition to the reduction in SB among the intervention group. This might indicate that standing has replaced some of the time spent in LPA and MVPA. Replacing sitting or reclining with standing has been shown to improve glucose and lipid metabolism by increasing skeletal muscle activity (54), and standing more has been shown to be beneficially associated with hepatic insulin sensitivity (55), which shows the importance of standing behavior in health promotion. However, the change in daily standing time during the twelve-month follow-up among the intervention group was only 18 min/day, which is not supposed to cause major improvements in health parameters, while it also replaces some of the daily MVPA and LPA, which are associated positively with cardiometabolic health (53, 54).

Altogether, MVPA and daily step count increased, and SB decreased during the six-month follow-up in both the intervention and control groups. It seems that participation alone in the present study increased the daily PA among the studied participants. However, the increases in the daily PA were not permanent, as the daily PA had decreased back to the baseline level in both groups by the time of the twelve-month follow-up. The LPA even decreased from the baseline values among the intervention group. Previous studies of PA interventions have shown similar results: the reduction of the achieved PA improvements back to the baseline levels after active monitoring has ended, even when statistically significant improvements in PA had been attained (24).

In terms of body composition, there was no group-by-time interaction during the follow-up. However, fat percentage declined during the twelve-month follow-up in both groups. PA increased from baseline to the six-month follow-up, but the PA had decreased back to the baseline level by the end of the twelve-month follow-up. This might indicate that changes in fat percentage and improvements in body composition can be due to other factors, such as diet (56) or improved sleep (57), while the application used in the intervention also provided feedback on sleeping habits.

Moreover, there was no group-by-time interaction regarding cardiometabolic biomarkers. Still, there was an increase in HbA1c, gluc, and INS, and a decrease in HDL during the follow-up in both groups. These findings indicate negative changes in both glucose and lipid metabolism. Further, these changes seemed not to be related to the changes in PA, and thus, they were probably due to other factors. Employees in high-stress occupations, such as firefighters, military, and law enforcement personnel, are exposed to a variety of stressors, such as psychological, physiological, and environmental stressors (58–60). These stressors have been shown to increase the risk of cardiovascular disease to develop (61). Cardiovascular risk factors for coronary disease and metabolic syndrome have been shown to be common among firefighters and police officers despite their good physical fitness levels (62). During shift work, there may often occur abnormal eating patterns caused by circadian stress (63), which may lead to consuming sugars, sodium, and saturated fat, which increase the prevalence of obesity, hyperlipidemia, and metabolic syndrome (63–65). While the FAT% decreased during the follow-up period, indicating decreased adiposity, increases in HbA1c, INS, and gluc, and a decrease in HDL might still be due to the quality of food consumed (66, 67).

Additionally, the results showed no group-by-time interaction regarding physical fitness, but the VO_2_max tended to increase in the intervention group compared to the control group. Fitness index and push-up results tended to increase during the twelve-month follow-up in both groups. While the intervention did not separate the type of PA (e.g., muscle strength training from aerobic exercise training), the intervention group participants were encouraged to perform, and positive changes in muscle strength could be expected. Yet, the accelerometers did not accurately recognize the movements, which are only performed with the upper or lower extremities (e.g., gym exercise) (68), so the changes in muscle strength were not expected to be remarkable. However, the number of results from the physical fitness tests was sparse at the six-month and twelve-month follow-ups, distorting the results.

There was no group-by-time interaction regarding experienced stress or work ability. The intervention group participants experienced less stress than the control group participants, and stress tended to increase during the intervention among the intervention group participants. Yet, there is no data from the control group after the intervention with which to compare these results, and therefore, the meaning of these findings remains unexplained concerning the differences between the groups.

This study has several limitations. The drop-out rate was 47%, which might be affected by the mobile nature of the work of soldiers. Soldiers working for the Finnish Defence Forces can be commanded to work in different places around Finland and might serve on crisis management missions in other countries at some point in their careers. Changing workplace to one in a different part of the country can lower the threshold for dropping out of the study, and being commanded to go on a crisis management mission would prevent continuing participation in the study. Added to this, the participantś retention of PA interventions tend to vary between 31% at the lowest to 100% at the highest (69), indicating that the drop-out rate of the current study is not unusual. Furthermore, the accelerometer was not water-resistant, so water activities could not be recorded. Also, the accelerometer may not accurately recognize movements that are only performed with the upper or lower extremities (e.g., gym exercises) and movements performed in a supine position (e.g., Pilates) (68). Moreover, it took two months to perform the baseline measurements, and the intervention could be started only after six months from the baseline measurements due to working schedules concerning conscriptś educational tasks. During these six months between the baseline measurements and the beginning of the intervention might have occurred changes in measured parameters. Further, the limitations include the lack of dietary assessment and the use of general advice to reduce SB and increase PA, despite having access to monitored behavioral data that could have allowed even more personalized recommendations.

The strengths of the study include the sensor-based measurements of the PA, measurement method taking advantage of accelerometers (70), which are also able to record separately LPA, MVPA, SB, and standing. Moreover, the created intervention takes advantage of smartphone technology, making it accessible to large populations, while smartphones or smart devices are commonly owned in the Western world. Furthermore, during the intervention, participants received constant individualized online feedback on their physical activity and sleeping habits, which extended throughout their workday and leisure time. This serves as an opportunity for precision prevention at both the workplace and in free time (26). Additionally, the created intervention needs only a little timely investment from the provider, while the only additional requirements besides a smartphone for the implementation of the intervention would be an accelerometer and consultation with a PA instructor once during the six-month period, neither of which requires a big investment of human resources.

In conclusion, the present intervention, utilizing accelerometer–smartphone app technology combined with telephone counseling and encouragement to use the possibility to exercise during two working hours a week, did not succeed in increasing PA, but it showed potential in increasing daily standing in the long term. Again, when comparing the intervention and control groups, there were no group-by-time interactions regarding body composition, cardiometabolic biomarkers, physical fitness, or work ability. However, there was an increase in stress among the intervention group during the intervention, which was not observed between baseline and the twelve-month follow-up. Also, VO_2_max tended to increase during the twelve-month follow-up among the intervention group participants; however the dropout rate was high in aerobic fitness tests, which likely biased the results. The study reveals the potential and challenges of a light PA intervention for large populations needing low investment time-wise by the provider, in terms of increasing PA and decreasing SB among military employees. From a practical point of view, a certain number of accelerometers could be provided to the willing employees, and physical education officers could arrange one PA counseling session every six months for them without these arrangements adding them massive workload. However, the variability of the weekly work environments, working hours, and work tasks challenges the implementation of this kind of PA intervention in a military workplace. Further, low adherence has been shown in device-based physical activity monitoring in the long term, and some studies have shown better achievements when the physical activity goals have been targeted by minutes (24, 71, 72). In potential future physical activity interventions, creating pre-scheduled time slots for exercise, also for field training weeks, targeting the physical activity goals by minutes and pre-scheduling the baseline and follow-up measurements, as well as the start of the intervention for set dates for all employees working in the brigades, would help to receive more reliable results. The authority responsible for planning the yearly schedules for the brigades could arrange the timetable so that it would not disturb the education of the conscripts. Further studies with larger sample sizes should be conducted to assess the effects of the intervention used in this study setting and the generalisability of the current findings.

## Data Availability

The data used in this study are not publicly available. According to the research permit granted by the Finnish Defence Command, the raw data is owned by the Finnish Defence Command. Further inquiries can be directed to the corresponding author.
