# Supplementary material for: A randomized controlled trial utilizing an interactive accelerometer linked to a smartphone application for enhancing physical activity and health among military employees
Source: Front Sports Act Living. 2025 Aug 22;7:1549980. doi: 10.3389/fspor.2025.1549980 (PMC12411429; doi:10.3389/fspor.2025.1549980)
Supplement: Supplementary file 1 [file Table1.docx]

**Supplementary Table A. Post-hoc power analysis.**

|  | Intervention group | | | Control group | | | Post-hoc power  (12 months) | | Difference between the groups ΔBL-6-months | Difference between the groups ΔBL-12-months |
| --- | --- | --- | --- | --- | --- | --- | --- | --- | --- | --- |
|  | Baseline  n=146 | 6 months  n=64 | 12 months  n=53 | Baseline  n=93 | 6 months  n=47 | 12 months  n=43 | Effect size | Power | t-test | t-test |
|  | Mean  (SD) | Mean  (SD) | Mean  (SD) | Mean  (SD) | Mean  (SD) | Mean  (SD) | Cohen’s d | % | p-value | p-value |
| **Physical activity** |  |  |  |  |  |  |  |  |  |  |
| Steps (number/day) | 6955 (2027) | 7628 (2231) | 6759  (1809) | 7237 (2250) | 8013 (2720) | 6972 (1989) | 0.112 | 8.4 | 0.304 | 0.883 |
| SB (min/day) | 560 (78) | 527 (84) | 544 (92) | 556 (93) | 520 (98) | 554 (1001) | 0.109 | 8.2 | 0.630 | 0.414 |
| MVPA (min/day) | 49 (20) | 56 (21) | 45 (17) | 52 (23) | 60 (32) | 48 (23) | 0.148 | 11.0 | 0.760 | 0.841 |
| LPA (min/day) | 221 (59) | 228 (56) | 215 (46) | 220 (57) | 231 (55) | 209 (48) |  |  | 0.271 | 0.895 |
| Standing (min/day) | 99 (41) | 110 (48) | 124 (60) | 112 (50) | 125 (59) | 120 (54) | 0.052 | 5.7 | 0.853 | 0.040 |
| **Physical fitness** |  |  |  |  |  |  |  |  |  |  |
| Fitness index | 3.5 (1.6)  n=127 | 4.2 (0.64)  n=12 | 3.7 (1.53)  n=14 | 3.5 (1.05)  n=87 | n=0 | 3.3 (1.17)  n=9 | 0.250 | 8.7 | - | 0.603 |
| VO2max (ml/kg/min) | 42.7 (9.5)  n=138 | 42.6 (9.0)  n=13 | 42.6 (10.8)  n=19 | 43.2 (13.2)  n=95 | n=0 | 44.5 (9.4)  n=10 | 0.184 | 7.4 | - | 0.056 |

Power calculations were performed using G*Power 3.1.9.
